# Racial differences in takotsubo cardiomyopathy outcomes in a large nationwide sample

**DOI:** 10.1002/ehf2.12664

**Published:** 2020-03-09

**Authors:** Raja Zaghlol, Amit K. Dey, Sameer Desale, Ana Barac

**Affiliations:** ^1^ Division of Internal Medicine Georgetown University/MedStar Washington Hospital Center Washington DC USA; ^2^ Section of Inflammation and Cardiometabolic Diseases National Heart, Lung and Blood Institute Bethesda MD USA; ^3^ Biostatistics and Biomedical Informatics Department MedStar Health Research Institute Hyattsville MD USA; ^4^ MedStar Heart and Vascular Institute MedStar Washington Hospital Center 110 Irving Street, NW, Ste 1218 Washington DC USA

**Keywords:** Takotsubo cardiomyopathy, Stress‐induced cardiomyopathy, Race, In‐hospital outcomes

## Abstract

**Aims:**

Takotsubo cardiomyopathy (TC) is characterized by transient ventricular impairment, often preceded by emotional or physical stress. Racial differences affect the outcomes of several cardiovascular conditions; however, the effect of race on TC remains unknown. This investigation aims to assess the effect of race on in‐hospital outcomes of TC in a large national sample.

**Methods and results:**

We conducted a US‐wide analysis of TC hospitalizations from 2006 to 2014 by querying the National Inpatient Sample database for the International Classification of Diseases‐ninth Revision TC code, characteristics, and inpatient outcomes. Patients with a primary diagnosis of acute coronary syndrome were excluded to reduce selection bias. Caucasians were compared with African Americans (AA) for differences in baseline characteristics and in‐hospital outcomes. Multivariate regression models were created to adjust for potential confounders. Of 97 650 TC patients, 83 807 (86.9%) were women, 89 624 (91.8%) identified as Caucasians, and 8026 (8.2%) as AA. The annual number of TC hospitalizations increased significantly from 2006 to 2014 in both races (from 335 to 21 265 annual cases, *P* < 0.001). In‐hospital mortality initially increased (1–2% in 2006 to 5–6% in 2009, *P* < 0.001) and subsequently remained relatively stable around 5–7% with no significant difference between races. In unadjusted analysis, AA had more cardiac arrests [304 (3.8%) vs. 2569 (2.9%), *P* = 0.04], invasive mechanical ventilation [1671 (20.8%) vs. 15 897 (17.7%), *P* = 0.002], tracheostomies [242 (3%) vs. 1600 (1.8%), *P* = 0.001], acute kidney injuries [1765 (22%) vs. 14 608 (16.3%), *P* < 0.0001], and longer hospital stays [4.5 (3.2–4.8) vs. 3.8 (3.7–3.9) days, *P* < 0.0001] compared with Caucasians. After the adjustment for differences in age, gender, comorbidities (using the enhanced Charlson comorbidity index), hospital location/teaching status, and socio‐economic factors, all differences were significantly attenuated or eliminated. Additionally, the adjusted risk was lower in AA compared with Caucasians, for cardiogenic shock [odds ratio (OR) 0.61 (0.47–0.78), *P* < 0.0001], mechanical ventilation [OR 0.8 (0.70–0.92), *P* = 0.002] and intraaortic balloon pump insertion [OR 0.63 (0.41–0.99), *P* = 0.04].

**Conclusions:**

Our investigation is the first large US‐wide analysis studying racial variations in TC outcomes. AA overall have more in‐hospital complications; however, the differences are driven by racial disparities in demographics, comorbidities, and socio‐economic factors.

## Introduction

Takotsubo cardiomyopathy (TC) or stress‐induced cardiomyopathy is characterized by transient ventricular impairment and regional wall hypokinesis, akinesis, or dyskinesis, often preceded by emotional or physical stressors.^1^ The syndrome presents similarly to acute myocardial infarction; however, the characteristic wall motion abnormality is not explained by the plaque rupture or obstructive coronary artery disease in the corresponding coronary artery territory.^2^ The pathophysiology of TC remains poorly understood although catecholamine excess has been proposed as an underlying mechanism^2^ and variability in individual susceptibility to catecholaminergic surge in the setting of acute and chronic stressors,^2^ modified by the pre‐existing medical and psychological conditions, has been suggested to affect presentation and outcomes of TC.^1^, ^3^, ^4^, ^5^, ^6^

Gender, racial, and ethnic differences have been reported with respect to incidence and prognosis of cardiovascular diseases including heart failure^7^ and acute myocardial infarction,^8^ in part due to variability in genetic, biological, and socio‐economic factors.^9^ Recent small studies have reported increased in‐hospital complications in patients of African American (AA) descent^10^, ^11^ presenting with TC; however, large population‐based studies are lacking. We investigated the effect of race on TC outcomes in a large nationwide sample of patients hospitalized the USA.

## Materials and methods

The National Inpatient Sample (NIS) database was used to obtain data on TC hospitalization and outcomes from 2006 to 2014. The NIS database is the largest publicly available all‐payer inpatient health database in the USA and was developed by the Agency for Healthcare Research and Quality for the Healthcare Cost and Utilization Project (HCUP). The database collects billing data from all states participating in the HCUP, representing over 97% of the US population.^12^ The NIS approximates a 20% stratified sample of discharges from US community hospitals covering over 7 million unweighted and 35 million weighted annual hospital stays.^12^

**Figure 1:**
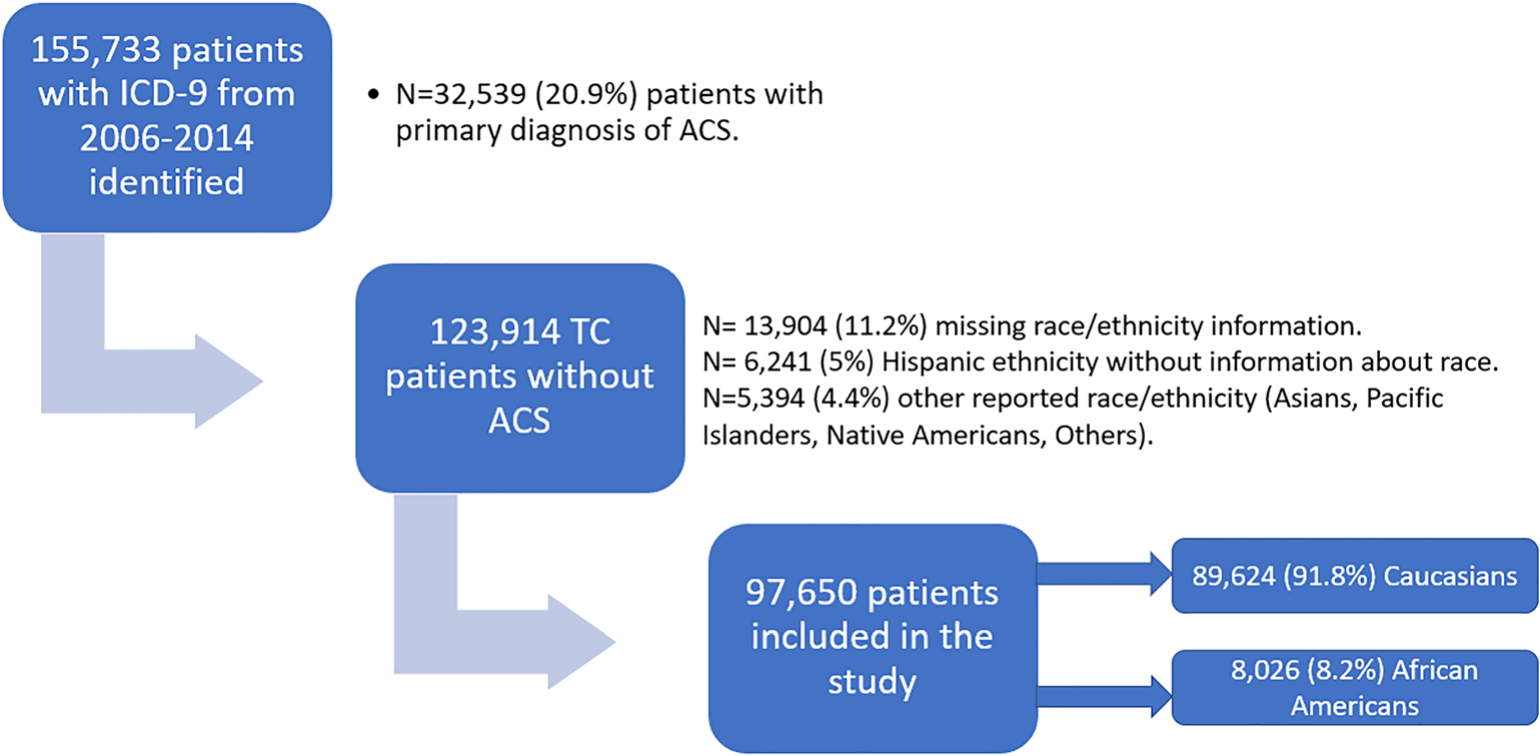
Patient selection. ICD‐9, International Classification of Diseases, Ninth Edition code; ACS, acute coronary syndrome; TC, takotsubo cardiomyopathy.

Patients over the age of 18 years hospitalized with The International Classification of Diseases‐ninth Revision (ICD‐9)‐Clinical Modification (ICD‐9‐CM) code 429.83 for TC from 2006 to 2014 were included in the study. We excluded patients with ICD‐9 codes for acute coronary syndrome (ACS) as their primary admitting diagnosis (*Table* *S1*). The analysis included patients with available self‐reported data about Caucasian (or White) and AA race (*Figure* 1). Patients who had a missing race or reported other race/ethnicity were excluded due to low prevalence (Asians, Pacific Islanders, and Native Americans) or confounding (Hispanic ethnicity, included in the NIS database under single race category, precluded accurate identification of race in these individuals). Weighted national estimates were obtained and used for the analysis using standard NIS methods.^13^

In‐hospital outcomes and complications (including cardiogenic shock, cardiac arrest, invasive mechanic ventilation, non‐invasive positive pressure ventilation, intraaortic balloon pump insertion, tracheostomy, acute kidney injury, length of stay, and mortality) were captured using the ICD‐9‐CM codes (*Table* *S2*). Outcomes of interest were selected based on prior published complications of TC either directly or due to the cardiogenic shock state observed in TC patients.^1^ Outcomes were adjusted for age, gender, baseline comorbidities using the enhanced Charlson comorbidity index (CCI)^14^ (*Table* *S3*), socio‐economic factors including smoking, drug abuse, household income, and primary insurance payer, as well as hospital type, location, and teaching status to account for potential confounders.

Continuous variables were reported as means and standard error of mean or as medians and interquartile range based on normality data. Categorical variables were reported as frequencies and percentages. Between‐group comparisons were conducted using t‐test, chi‐square test and signed‐rank test. Multivariate regression models were used to adjust for potential confounders. Adjusted outcomes were reported as odds ratio (OR) with 95% confidence interval (CI). SAS® version 9.4 software was used for data analysis and *P*‐value <0.05 (two‐tailed) was considered statistically significant.

The Institutional Review Board (IRB) exemption was approved for this analysis based on publicly available NIS datasets that contain de‐identified patient information. All authors with access to the data completed and signed the HCUP data use agreement training course.

## Results

From 2006 to 2014, 155 733 hospitalizations with diagnosis of TC in any diagnostic field were identified. Patients with primary diagnosis of ACS (*N* = 32 539) and missing race/ethnicity information (*N* = 13 904) were excluded (*Figure* 1). We also excluded patients with reported Hispanic ethnicity because of the lack of information about the race (*N* = 6241) and other races (*N* = 5394) because of low prevalence. Of 97 650 patients included, 83 807 (86.9%) were women, 89 624 (91.8%) identified as Caucasian, and 8026 (8.2%) as AA. Clinical and demographic characteristics and hospital and insurance variables were all significantly different between the two races, with AA being younger at presentation, having higher prevalence of men, higher CCI, and lower median household income (*Table* 1).

**Table 1:** Demographic and clinical characteristics

Parameter	Caucasians (*n* = 89 624)	Race African Americans (*n* = 8026)	Total (*N* = 97 650)	*P*‐value
Age, years mean (SEM)	67.5 (0.13)	61.8 (0.45)	67 (0.14)	<0.0001
Gender, *n* (%)				
Female	78 135 (87.2)	6672 (83.1)	84 807 (86.9)	<0.0001
Past medical history, *n* (%)				
Hypertension	55 926 (62.4)	5651 (70.4)	61 577 (63.1)	<0.0001
Congestive heart failure	20 693 (23.1)	2066 (25.7)	22 760 (23.3)	0.03
Chronic lung disease	26 815 (29.9)	1971 (24.5)	28 786 (29.5)	<0.0001
Diabetes mellitus without complications	15 295 (17.1)	2071 (25.8)	17 366 (17.8)	<0.0001
Diabetes mellitus with complications	2577 (2.9)	446 (5.6)	3023 (3.1)	<0.0001
Chronic kidney disease	9065 (10.1)	1315 (16.4)	11 174 (10.8)	<0.0001
Depression	16 149 (18)	930 (11.6)	17 079 (17.5)	<0.0001
Psychotic disorders	5111 (5.7)	432 (5.4)	5543 (5.7)	0.61
Smoking	29 535 (32.9)	2660 (33.1)	6211 (6.4)	0.90
Drug abuse	3642 (4.06%)	613 (7.64%)	4255 (4.4)	<0.0001
Charlson comorbidity index, *n* (%)				
0–1	37 950 (42.3)	2675 (33.3)	40 625 (41.6)	<0.0001
2–3	34 229 (38.2)	3186 (39.7)	37 415 (38.3)	
≥4	17 445 (19.5)	2169 (27)	19 614 (20.1)	
Hospital location/teaching status, *n* (%)				
Rural	6473 (7.3)	199 (2.5)	6672 (6.9)	<0.0001
Urban non‐teaching	30 548 (34.3)	1414 (17.7)	31,962 (33)	
Urban teaching	52 158 (58.5)	6366 (79.8)	58 523 (60.2)	
Region, *n* (%)				
Northeast	19 546 (21.8)	1710 (21.3)	21 256 (21.8)	<0.0001
Midwest	20 089 (22.4)	2047 (25.5)	22 136 (22.7)	
South	30 398 (33.9)	3277 (40.8)	33 675 (34.5)	
West	19 591 (21.9)	996 (12.4)	20 587 (21.1)	
Primary payer, *n* (%)				
Medicare	57 442 (64.2)	4,147 (51.7)	61 589 (63.1)	<0.0001
Medicaid	5723 (6.4)	1,437 (17.9)	7160 (7.3)	
Private	21 546 (24.1)	1,791 (22.3)	23 337 (23.9)	
Self‐pay	2888 (3.2)	413 (5.2)	3301 (3.4)	
No charge/other	1927 (2.2)	233 (2.9)	2160 (2.2)	
Median household income, *n* (%)				
Q1	18 833 (21.4)	3983 (50.4)	22 816 (23.8)	<0.0001
Q2	22 512 (25.5)	1626 (20.6)	24 138 (25.1)	
Q3	22 948 (26)	1395 (17.7)	24 343 (25.3)	
Q4	23 864 (27)	892 (11.3)	24 757 (25.8)	

*n*: number; Q, income quartile; SEM, standard error of the mean.

**Figure 2:**
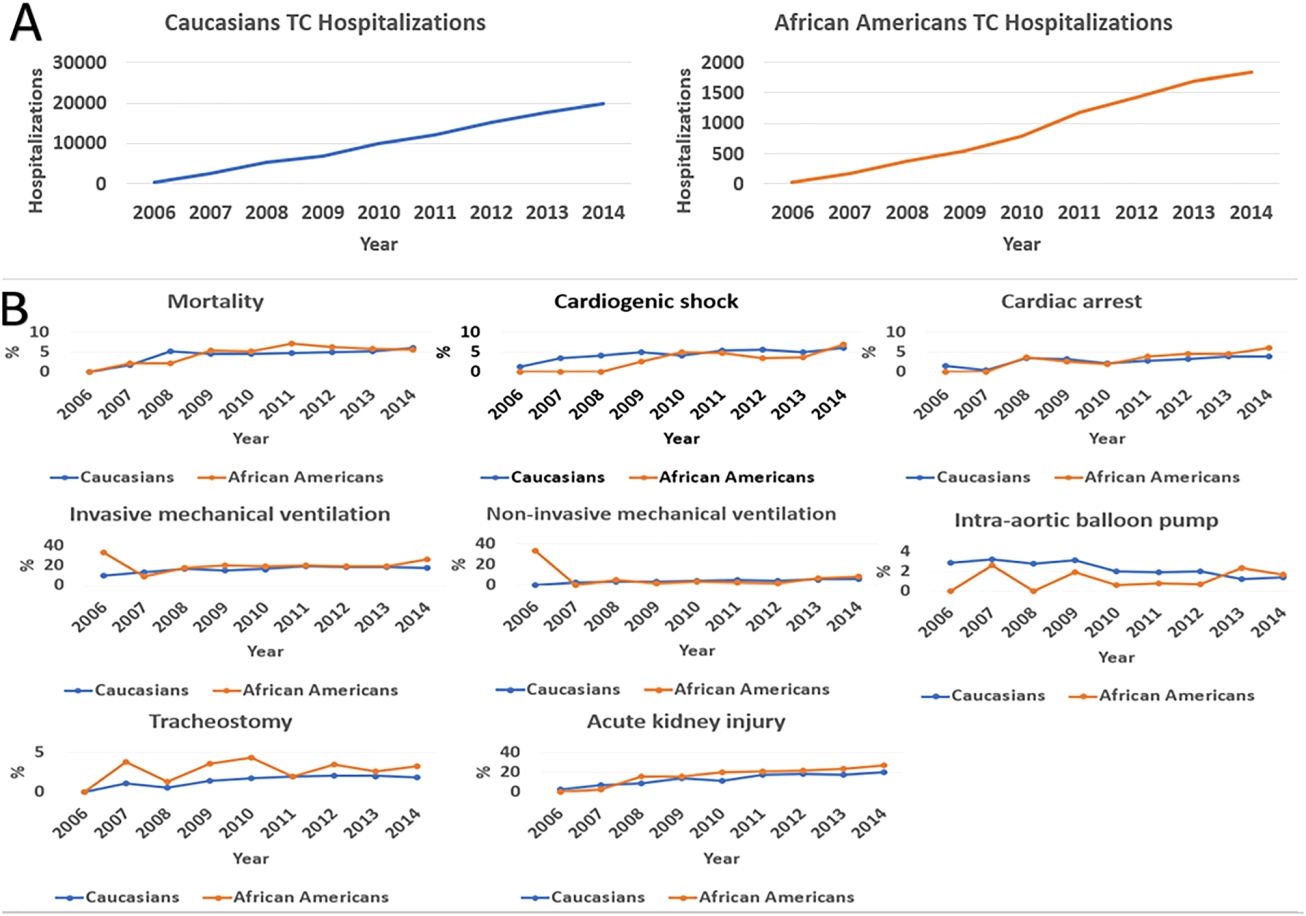
Takotsubo cardiomyopathy trends for hospitalization (A) and unadjusted in‐hospital outcomes (B). TC, takotsubo cardiomyopathy.

Annual trends for TC hospitalizations and in‐hospital outcomes are shown in *Figure* 2. The rate of TC hospitalizations increased from 2006 to 2014 in both races (from 335 to 21 265 annual cases) (*Figure* 2 *A*). Mortality rates initially increased [from 50 cases (1–2%) in 2006 to 340 cases (5–6%) in 2009, *P* < 0.001] and subsequently remained relatively stable around 5–7% with annual fluctuations but with no overall significant difference between races (*Figure* 2 *B*).

**Figure 3:**
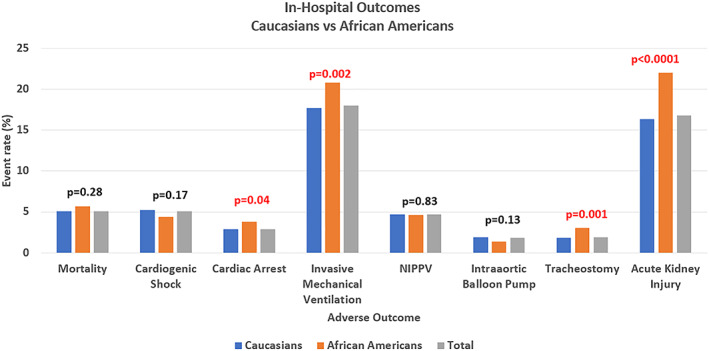
Unadjusted comparison of in‐hospital outcomes. NIPPV, non‐invasive positive pressure ventilation.

We then compared in‐hospital outcomes and complications between Caucasians and AAs (*Figure* 3). In unadjusted analysis, AAs had more cardiac arrests [304 (3.8%) vs. 2569 (2.9%), *P* = 0.04], invasive mechanical ventilation [1671 (20.8%) vs. 15 897 (17.7%), *P* = 0.002], tracheostomies [242 (3%) vs. 1600 (1.8%), *P* = 0.001], and acute kidney injuries [1765 (22%) vs. 14 608 (16.3%), *P* < 0.0001] compared with Caucasians. AA also had longer hospital stay [median of 4.5 (3.2–4.8) vs. 3.8 (3.7–3.9) days, *P* < 0.0001].

The ORs between potential confounders, including age, gender and comorbidities, and outcomes are shown in *Table* *S4*. Age was not significantly associated with any of the in‐hospital outcomes; however, male gender strongly predicted mortality (OR 2.2, 95% CI 1.9–2.6), cardiogenic shock (OR 1.4, 95% CI 1.2–1.7), cardiac arrests (OR 1.6, 95% CI 1.32–1.96), mechanical ventilation (OR 1.7, 95% CI 1.5–1.9), intraaortic balloon pump (OR 1.4, 95% CI 1–1.8), tracheostomy (OR 1.8, 95% CI 1.4–2.3), and acute kidney injury (OR 2, 95% CI 1.8–2.2). Similarly, more comorbidities evident by higher CCI were strongly associated with all captured outcomes (*Table* *S4*).

Three adjustment models that account for differences in age, gender, comorbidities, hospital location/teaching status, and socio‐economic factors are shown in *Table* 2. In Model 1, after adjustment for age and gender, differences in cardiac arrest and mechanical ventilation were no longer significant. In Models 2 and 3, differences in tracheostomy and acute kidney injury were progressively attenuated to eliminated by the adjustment for CCI, hospital location/teaching status (tracheostomy), and psychiatric conditions/socio‐economic factors (acute kidney injury). Together, AA race was not associated with worse in‐hospital outcomes when adjusted for both comorbidities and socio‐economic factors. In contrast, cardiogenic shock, not significantly different between the races in unadjusted analysis, was more prevalent among Caucasians after the adjustments (*Table* 2). Similarly, in fully adjusted analysis, Caucasians had more mechanical ventilation and intraaortic balloon pump insertions.

**Table 2:** In‐hospital outcomes: adjusted comparisons between African Americans and Caucasians

Outcome	Model 1*	*P*‐value	Model 2†	*P*‐value	Model 3‡	*P*‐value
Mortality	1.14 (0.90–1.43)	0.27	0.94 (0.74–1.19)	0.62	0.86 (0.67–1.1)	0.21
Cardiogenic shock	0.75 (0.59–0.95)	0.02	0.63 (0.50–0.8)	0.0001	0.58 (0.46–0.75)	<0.0001
Cardiac arrest	1.06 (0.81–1.37)	0.69	1 (0.76–1.3)	0.99	0.96 (0.73–1.27)	0.77
Mechanical ventilation	1.02 (0.89–1.16)	0.78	0.86 (0.76–0.98)	0.02	0.78 (0.68–0.9)	0.001
NIPPV	1.05 (0.81–1.36)	0.70	0.90 (0.69–1.17)	0.43	0.88 (0.67–1.17)	0.37
Intraaortic balloon pump	0.64 (0.41–0.98)	0.04	0.57 (0.37–0.89)	0.013	0.6 (0.39–0.94)	0.02
Tracheostomy	1.43 (1.02–2.01)	0.04	1.08 (0.77–1.53)	0.65	1.1 (0.75–1.54)	0.70
Acute kidney injury	1.49 (1.30–1.70)	<0.0001	1.23 (1.07–1.41)	0.003	1.16 (1.0–1.34)	0.05

Data are presented as African Americans compared to Caucasians with odds ratio (OR) and 95% confidence interval.

NIPPV, non‐invasive positive pressure ventilation; OR, odds ratio.

* Adjusted for age and gender.

† Adjusted for age, gender, comorbidities, and hospital location/teaching status.

‡ Adjusted for age, gender, comorbidities, hospital location/teaching status, psychiatric conditions (depression and psychosis), and socio‐economic factors including smoking, drug abuse, household income, and primary insurance payer.

## Discussion

In a US‐wide sample of hospitalized patients with TC, we report the following novel observations: (i) The temporal incidence in TC hospitalizations from 2004 to 2016 has increased with stable mortality rates in Caucasians and AA, (ii) AA overall have worse outcomes including longer hospitalizations, higher rates of cardiac arrests, invasive mechanical ventilation, tracheostomies, and acute kidney injury compared with Caucasians, (iii) In fully adjusted analysis, AA race was not associated with worse in‐hospital outcomes, suggesting that the racial differences observed were driven by the higher prevalence of comorbidities and adverse socio‐economic profile among AA. After adjustment for those confounders, the differences in outcomes are attenuated and/or longer significant.

Our results are consistent with prior reports of a marked increase in hospitalization for TC in the USA,^1^, ^15^ and provide new data on racial trends, showing similar annual increase in the number of TC hospitalization among Caucasians and AA. While it is possible that there has been an actual increase in TC occurrence, the observed numbers are more likely due to better recognition of the syndrome worldwide.^1^ Prior studies showed that despite higher incidence and better recognition of the syndrome, short‐term mortality rates remained relatively high, matching that of acute myocardial infarction.^1^ Similarly, in our analysis, we found mortality rates in the range of 5–7% with annual fluctuations, but no overall difference between the races. We hypothesize that limited understanding of the pathophysiology of the disease, absence of risk stratification tools, and lack of standards in management may be contributing to high mortality.^16^

Our observations of worse in‐hospital complications among AA patients with TC, including longer hospitalization, higher rates of mechanical ventilation, cardiac arrests, and acute kidney injury, confirm our prior findings from a small single‐centre cohort.^4^, ^11^ Similarly, in a two‐centre analysis Dias *et al*. reported worse TC outcomes in AA with longer hospitalizations, more mechanical ventilation, lower left ventricular ejection fraction on presentation, and higher cardiac troponin elevation compared with Caucasians.^10^ Our study builds on the prior reports by allowing adjusted analysis to include differences in age, gender, medical comorbidities, and socio‐economic factors. While age was not significantly associated with outcomes in the overall cohort, male gender was a strong predictor of mortality, cardiogenic shock, and cardiac arrest as well as majority of other adverse outcomes, similar to prior reports^6^, ^17^, ^18^, ^19^. In our cohort, AA had a significantly higher proportion of men, which likely contributed to the observed differences. Indeed, after the adjustment for gender, AA race was significantly associated only with higher rates of tracheostomy and acute kidney injury although with attenuation of OR. To our knowledge, this is the first report of the worse TC outcomes among AA men as an important contributor to the observed racial differences. The observed worse outcomes in AA could be due to higher proportion of male gender in that cohort, or to a potential gender–race interaction where TC affects AA men more severely than Caucasian men, which will be explored in future study. The mechanisms underlying observed racial differences have not been elucidated, however, variations in genes regulating sympathetic activity as well as differences in endothelial homeostasis have been described. Kurnik *et al*. demonstrated increased hemodynamic sympathetic response in AA after noxious stimuli compared with Caucasians, which was correlated with racial variation in polymorphisms in the genes encoding alpha2C‐adrenergic receptor and G‐protein beta3‐subunit.^20^ AA were reported to have increased susceptibility to endothelial injury by enhanced nitric oxide inactivation,^21^ possibly predisposing them to coronary microcirculatory dysfunction, one of the mechanisms suggested to play role in the pathogenesis of acute TC.^22^, ^23^ Similarly, Campia *et al*. reported reduced responsiveness of conduit vessels to both endogenous and exogenous nitric oxide in AA^24^ and Ergul *et al*. reported higher plasma endothelin‐1 level in AA^25^ compared with Caucasians.

Additional adjustment for differences in comorbidities and socio‐economic factors further attenuated the association between AA and worse tracheostomy and acute kidney injury outcomes. It is plausible that presence of chronic medical conditions, reflected in CCI, and lower socio‐economic status may lead to adverse TC outcomes via increased levels of chronic stress. In our cohort, AA had significantly lower median household income than Caucasians, with about half of AA falling below the first income quartile, as compared with only 21% of Caucasians. AA also had higher prevalence of severe chronic medical conditions measured by a significantly higher CCI (CCI ≥ 4 in 27% in AA vs. 19.5% in Caucasians, *P* < 0.0001). These observations open a hypothesis whether worse outcomes among AA may be in part explained by higher level of chronic, medical, and socio‐economic stressors and thus, possibly higher catecholamines. Among socio‐economic factors, substance abuse disorders have been reported more prevalent among AA,^26^ and our study confirmed this association with higher rates of drug abuse in AA as compared with Caucasians (7.6% vs. 4%, *P* < 0.0001). Stimulants such as cocaine and amphetamines increase sympathetic outflow and were reported as potential causes of TC.^27^, ^28^ Similarly, alcohol and opioid withdrawals were reported to trigger TC due to catecholamine surges.^29^, ^30^ Thus, higher rates of substance abuse among AA may predispose them to chronically elevated levels of catecholamines resulting in worse TC outcomes.

In fully adjusted analysis, accounting for age, gender, comorbidities, hospital location/teaching status, and socio‐economic factors, the likelihood of mechanical ventilation, cardiogenic shock, and intraaortic balloon pump use was higher among Caucasians. Although those findings are novel, they should be interpreted with caution. Given those observations appeared only after adjustment for several confounders, studies investigating the interaction effect between race and those confounders are needed before attributing worse outcomes to true racial effect.

The prevalence of AA race in our studied TC population was slightly lower than the general population of the USA (8.2% vs. 12.6% respectively^31^). Although this may suggest lower TC incidence in blacks, it may be due to multiple complex factors, which may need to be studied in a prospective fashion. First, the random sampling method used in the NIS may potentially underestimate disease prevalence in minorities. This can be due to unequal distribution of minority populations across different states of the USA. Nevertheless, this remains the largest and most representative national sample of AA patients with TC reported to date. Second, this may be due to underdiagnosis of TC in AA because various socio‐economic and medical factors such as low household income and high burden of comorbid conditions among AA may limit their ability to receive high quality health care, increasing the likelihood of missed diagnosis. This was previously demonstrated with other cardiovascular conditions such as congestive heart failure and coronary artery disease.^32^ The strengths of our study include large sample size, inclusion, and balanced representation of community and tertiary hospitals across the USA, reducing the effect of potential geographic confounders. The exclusion of patients with a primary diagnosis of ACS (a common potential mimic of TC) reduced selection bias. High event rate allowed us to adjust for medical and socio‐economic confounders. Limitations of our study include retrospective design and inability to verify the accuracy of diagnoses or outcomes that were based on ICD‐9 codes. The NIS database captures hospitalizations rather than individual patients; thus, some of the captured hospitalizations may be recurrences in the same subject rather than a different patient. However, the reported recurrence rate of TC in the literature is less than 5%, making it unlikely to affect our results.^33^, ^34^ Finally, we did not include patients diagnosed with TC concomitantly with or secondary to ACS because of concern for diagnosis bias. The recognition of this entity is fairly recent and is dependent on differentiating regional wall motion abnormalities caused by obstructive coronary artery disease from TC. This distinction relies heavily on coronary angiograms and echocardiograms, which are unavailable in the NIS.^1^, ^35^

In conclusion, in this multicentre, large, population analysis of patients hospitalized with TC, AA had worse in‐hospital outcomes compared with Caucasians. The observed differences were mostly driven by higher prevalence of male gender among AA in addition to differences in medical and socio‐economic factors. Our study provides a rationale for further investigations of differences in TC mechanisms and outcomes among men and women of different races and development of risk stratification models.

## Conflict of interest

All other authors declare no conflicts of interests in relation to the work presented in this manuscript.

## Supporting information


**Supplemental Table 1:** ICD9‐CM codes for acute myocardial infarction that were excluded from the study population.
**Supplemental table 2:** ICD‐9 CM codes, and comorbidity codes used in defining the cohort, comorbidities and complications.
**Supplemental Table 3:** ICD‐9‐CM codes used by comorbidity to compute the Charlson comorbidity index.
**Supplemental table 4:** Effect of age, gender and comorbidities on TC in‐hospital outcomes.
