# Supplementary material for: Racial differences in takotsubo cardiomyopathy outcomes in a large nationwide sample
Source: ESC Heart Fail. 2020 Mar 9;7(3):1056–63. doi: 10.1002/ehf2.12664 (PMC7261569; doi:10.1002/ehf2.12664)
Supplement: Supplementary file 1 — Supplemental Table 1: ICD9‐CM codes for acute myocardial infarction that were excluded from the study population. Supplemental table 2: ICD‐9 CM codes, and comorbidity codes used in defining the cohort, comorbidities and complications. Supplemental Table 3: ICD‐9‐CM codes used by comorbidity to compute the Charlson comorbidity index. Supplemental table 4: Effect of age, gender and comorbidities on TC in‐hospital outcomes. [file EHF2-7-1056-s001.docx]

**Supplementary Data:**

**Supplemental Table 1:** ICD9-CM codes for acute myocardial infarction that were excluded from the study population.

| **Variable** | **Codes** |
| --- | --- |
| Acute myocardial infarction of anterolateral wall, episode of care unspecified | 410.00 |
| Acute myocardial infarction of anterolateral wall, initial episode of care | 410.01 |
| Acute myocardial infarction of anterolateral wall, subsequent episode of care | 410.02 |
| Acute myocardial infarction of other anterior wall, episode of care unspecified | 410.10 |
| Acute myocardial infarction of other anterior wall, initial episode of care | 410.11 |
| Acute myocardial infarction of other anterior wall, subsequent episode of care | 410.12 |
| Acute myocardial infarction of inferolateral wall, episode of care unspecified | 410.20 |
| Acute myocardial infarction of inferolateral wall, initial episode of care | 410.21 |
| Acute myocardial infarction of inferolateral wall, subsequent episode of care | 410.22 |
| Acute myocardial infarction of inferoposterior wall, episode of care unspecified | 410.30 |
| Acute myocardial infarction of inferoposterior wall, initial episode of care | 410.31 |
| Acute myocardial infarction of inferoposterior wall, subsequent episode of care | 410.32 |
| Acute myocardial infarction of other inferior wall, episode of care unspecified | 410.40 |
| Acute myocardial infarction of other inferior wall, initial episode of care | 410.41 |
| Acute myocardial infarction of other inferior wall, subsequent episode of care | 410.42 |
| Acute myocardial infarction of other lateral wall, episode of care unspecified | 410.50 |
| Acute myocardial infarction of other lateral wall, initial episode of care | 410.51 |
| Acute myocardial infarction of other lateral wall, subsequent episode of care | 410.52 |
| True posterior wall infarction, episode of care unspecified | 410.60 |
| True posterior wall infarction, initial episode of care | 410.61 |
| True posterior wall infarction, subsequent episode of care | 410.62 |
| Subendocardial infarction, episode of care unspecified | 410.70 |
| Subendocardial infarction, initial episode of care | 410.71 |
| Subendocardial infarction, subsequent episode of care | 410.72 |
| Acute myocardial infarction of other specified sites, episode of care unspecified | 410.80 |
| Acute myocardial infarction of other specified sites, initial episode of care | 410.81 |
| Acute myocardial infarction of other specified sites, subsequent episode of care | 410.82 |
| Acute myocardial infarction of unspecified site, episode of care unspecified | 410.90 |
| Acute myocardial infarction of unspecified site, initial episode of care | 410.91 |
| Acute myocardial infarction of unspecified site, subsequent episode of care | 410.92 |
| Intermediate coronary syndrome | 411.10 |
| Acute coronary occlusion without myocardial infarction | 411.81 |

**Supplemental table 2:** ICD-9 CM codes, and comorbidity codes used in defining the cohort, comorbidities and complications.

| **Variable** | **Codes** | **Comorbidity field from NIS** |
| --- | --- | --- |
| Takotsubo Cardiomyopathy | 429.83 |  |
| **Comorbidities** |  |  |
| Congestive heart failure | 425.xx | CM_CHF |
| Hypertension |  | CM_HTN_C |
| Diabetes |  | CM_DX, CM_DMCX |
| Smoking | 305.1x, V158.2 |  |
| Chronic renal disease |  | CM_RENLFAIL |
| Chronic lung disease |  | CM_CHRNLUNG |
| Drug abuse |  | CM_DRUG |
| **In-Hospital Complications and Dispositions** |  |  |
| Acute Kidney Injury | 584.xx |  |
| Cardiogenic Shock | 785.51 |  |
| Cardiac Arrest | 427.50 |  |
| Invasive Mechanical Ventilation | 96.70 |  |
| Non-Invasive Mechanical Ventilation | 93.90 |  |
| Implant of Pulsatile Balloon | 37.61 |  |
| Tracheostomy | 31.10, 31.21, 31.29 |  |

**Supplemental Table 3:** ICD-9-CM codes used by comorbidity to compute the Charlson comorbidity index.

| **Variable** | **Codes** | **Points** |
| --- | --- | --- |
| Myocardial infarction | 410.x, 412.x | 1 Point |
| Congestive heart failure | 398.91, 402.01, 402.11, 402.91, 404.01, 404.03, 404.11, 404.13, 404.91, 404.93, 425.4 - 425.9, 428.x | 1 Point |
| Peripheral vascular disease | 093.0, 437.3, 440.x, 441.x, 443.1 - 443.9, 47.1, 557.1, 557.9, V43.4 | 1 Point |
| Cerebrovascular disease | 362.34, 430.x - 438.x | 1 Point |
| Dementia | 290.x, 294.1, 331.2 | 1 Point |
| Chronic pulmonary disease | 416.8, 416.9, 490.x - 505.x, 506.4, 508.1, 508.8 | 1 Point |
| Rheumatic disease | 446.5, 710.0 - 710.4, 714.0 - 714.2, 714.8, 725.x | 1 Point |
| Peptic ulcer disease | 531.x - 534.x | 1 Point |
| Mild liver disease | 070.22, 070.23, 070.32, 070.33, 070.44, 070.54, 070.6, 070.9, 570.x, 571.x, 573.3, 573.4, 573.8, 573.9, V42.7 | 1 Point |
| Diabetes without chronic complication | 250.0 - 250.3, 250.8, 250.9 | 1 Point |
| Diabetes with chronic complication | 250.4 - 250.7 | 2 Points |
| Hemiplegia or paraplegia | 334.1, 342.x, 343.x, 344.0 - 344.6, 344.9 | 2 Points |
| Renal disease | 403.01, 403.11, 403.91, 404.02, 404.03, 404.12, 404.13, 404.92, 404.93, 582.x, 583.0 - 583.7, 585.x, 586.x, 588.0, V42.0, V45.1, V56.x | 2 Points |
| Any malignancy, including lymphoma and leukemia, except malignant neoplasm of skin | 140.x - 172.x, 174.x - 195.8, 200.x - 208.x, 238.6 | 2 Points |
| Moderate or severe liver disease | 456.0 - 456.2, 572.2- 572.8 | 3 Points |
| Metastatic solid tumor | 196.x - 199.x | 6 Points |
| Human Immunodeficiency Virus Infection | 042.x - 044.x | 6 Points |

**Supplemental table 4:** Effect of age, gender and comorbidities on TC in-hospital outcomes.

| **Outcome** | **Age** | **Gender (Male vs Female)** | **CCI (2-3 vs 0-1)** | **CCI (4+ vs 0-1)** |
| --- | --- | --- | --- | --- |
| **Mortality** | 1.00 (1.0-1.01) | 2.20 (1.88-2.57) | 1.74 (1.48-2.06) | 3.60 (3.03-4.27) |
| **Cardiogenic shock** | 0.98 (0.98-0.99) | 1.43 (1.21-1.67) | 2.12 (1.81-2.49) | 2.50 (2.09-2.99) |
| **Cardiac Arrest** | 0.97 (0.97-0.98) | 1.61 (1.32-1.96) | 1.39 (1.16-1.66) | 1.30 (1.04-1.61) |
| **Mechanical ventilation** | 0.97 (0.96-0.97) | 1.69 (1.53-1.86) | 2.35 (2.16-2.57) | 3.14 (2.84-3.48) |
| **NIPPV** | 1.00 (1.00-1.01) | 1.05 (0.89-1.28) | 3.48 (2.88-4.2) | 4.14 (3.36-5.10) |
| **Intraaortic balloon pump** | 0.98 (0.97-0.99) | 1.35 (1.02-1.79) | 1.76 (1.37-2.25) | 1.54 (1.15-2.06) |
| **Tracheostomy** | 0.98 (0.97-0.98) | 1.77 (1.38-2.27) | 2.1 (1.60-2.75) | 3.49 (2.61-4.66) |
| **Acute kidney injury** | 1.00 (0.99-1.00) | 1.99 (1.79-2.22) | 2.07 (1.87-2.28) | 4.76 (4.28-5.29) |

TC: takotsubo cardiomyopathy; NIPPV: non-invasive positive pressure ventilation; CCI: Charlson comorbidity index.
Data are reported as odds ratio (OR) and 95% confidence intervals.
